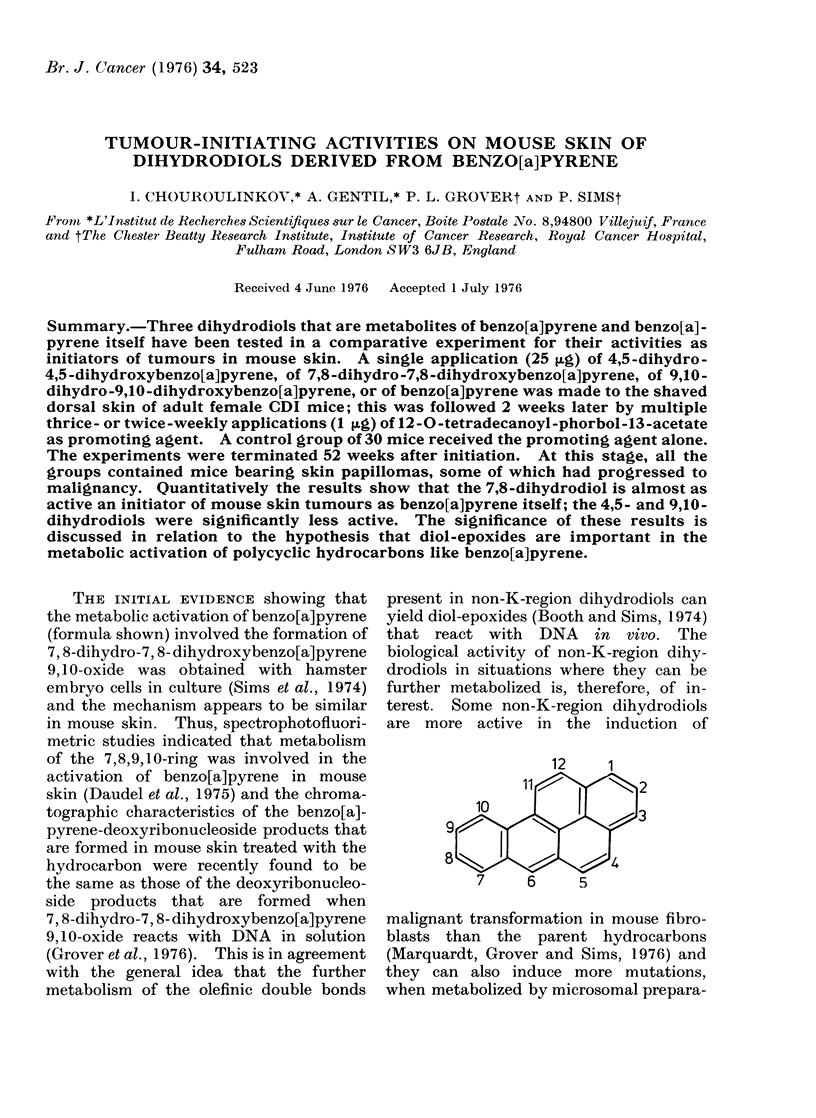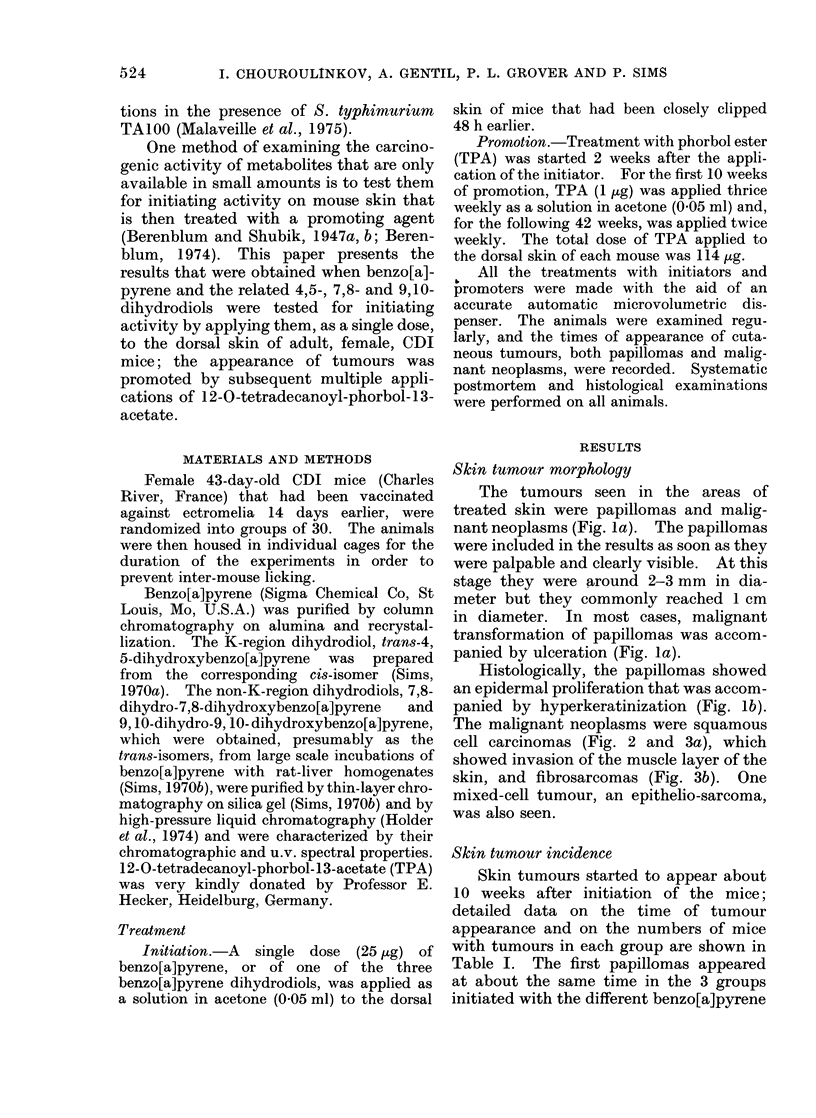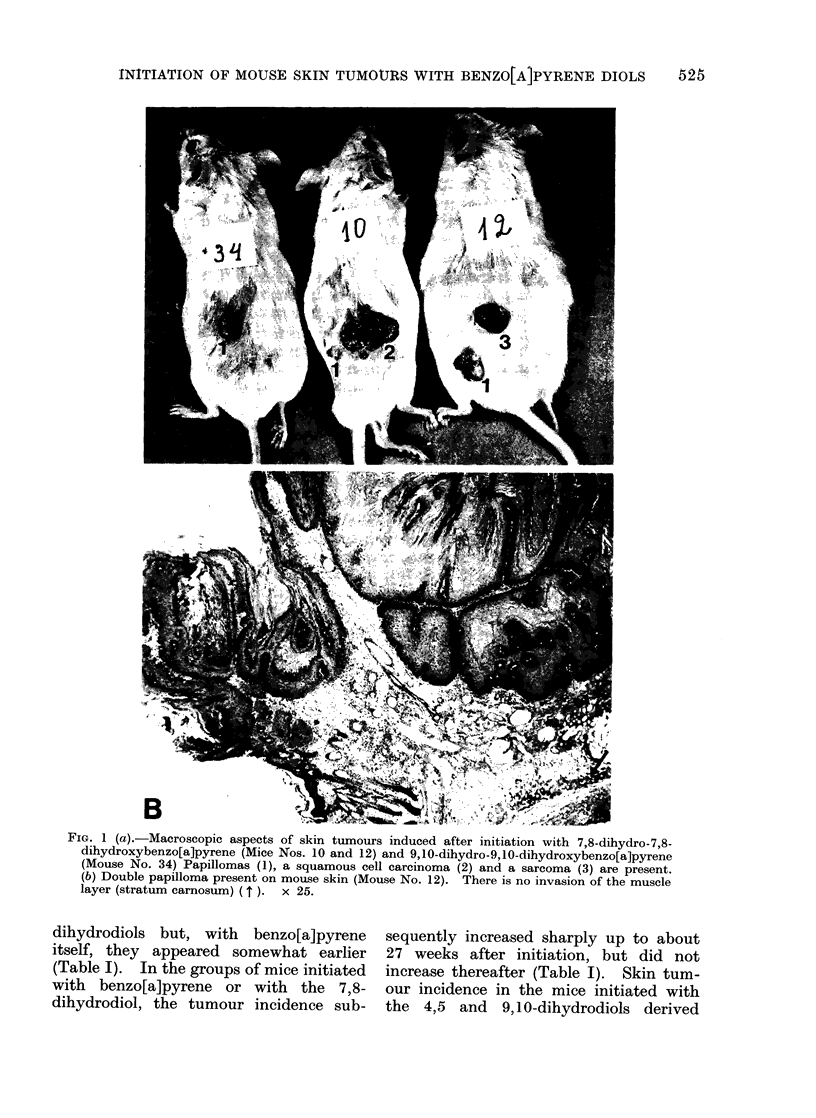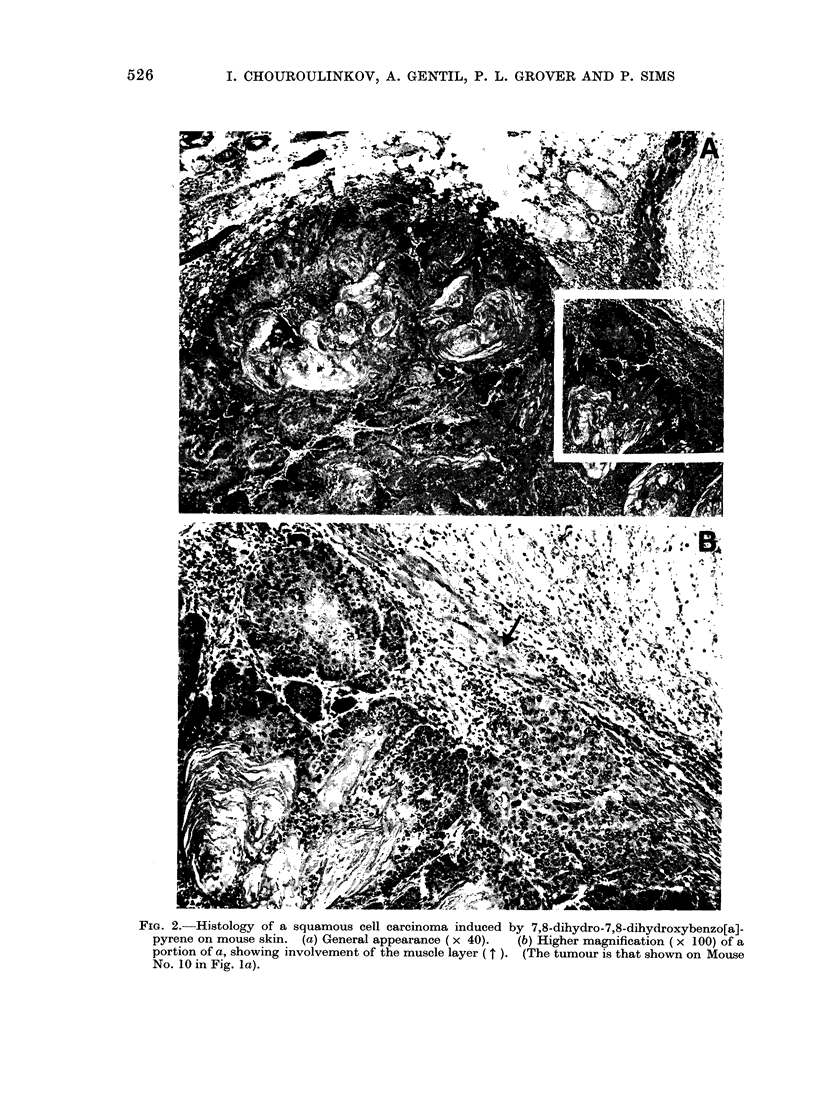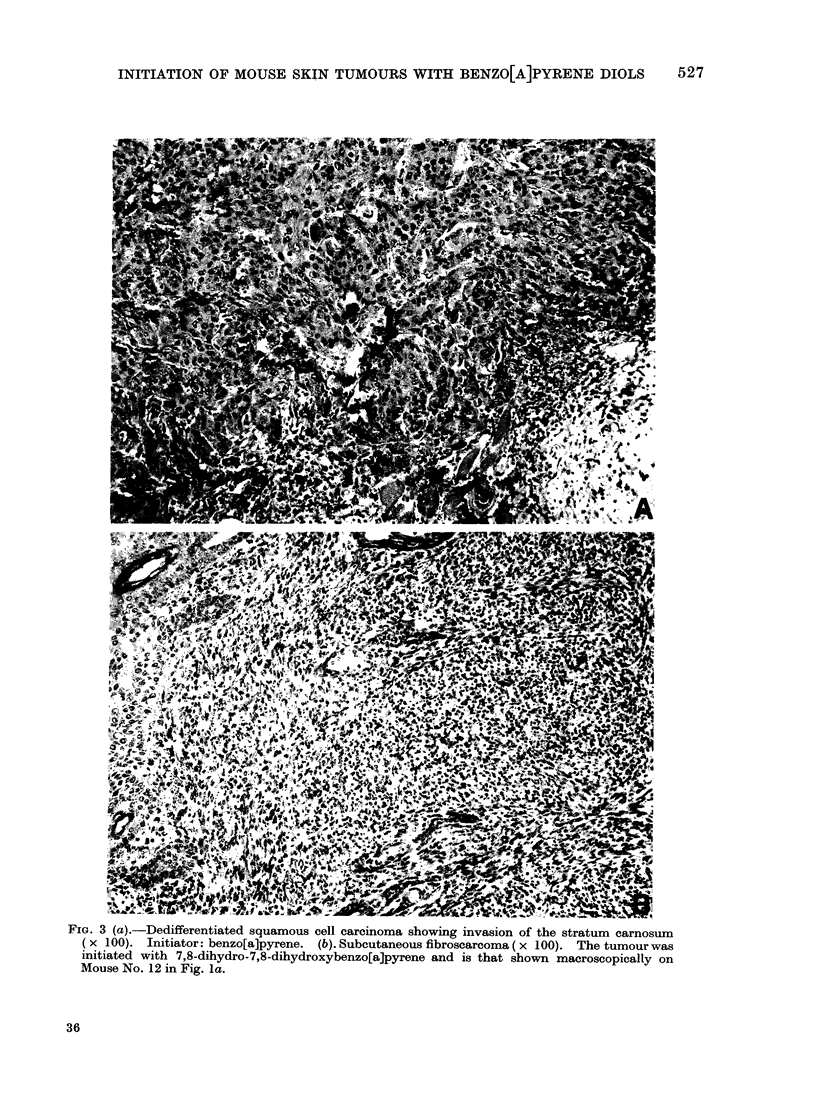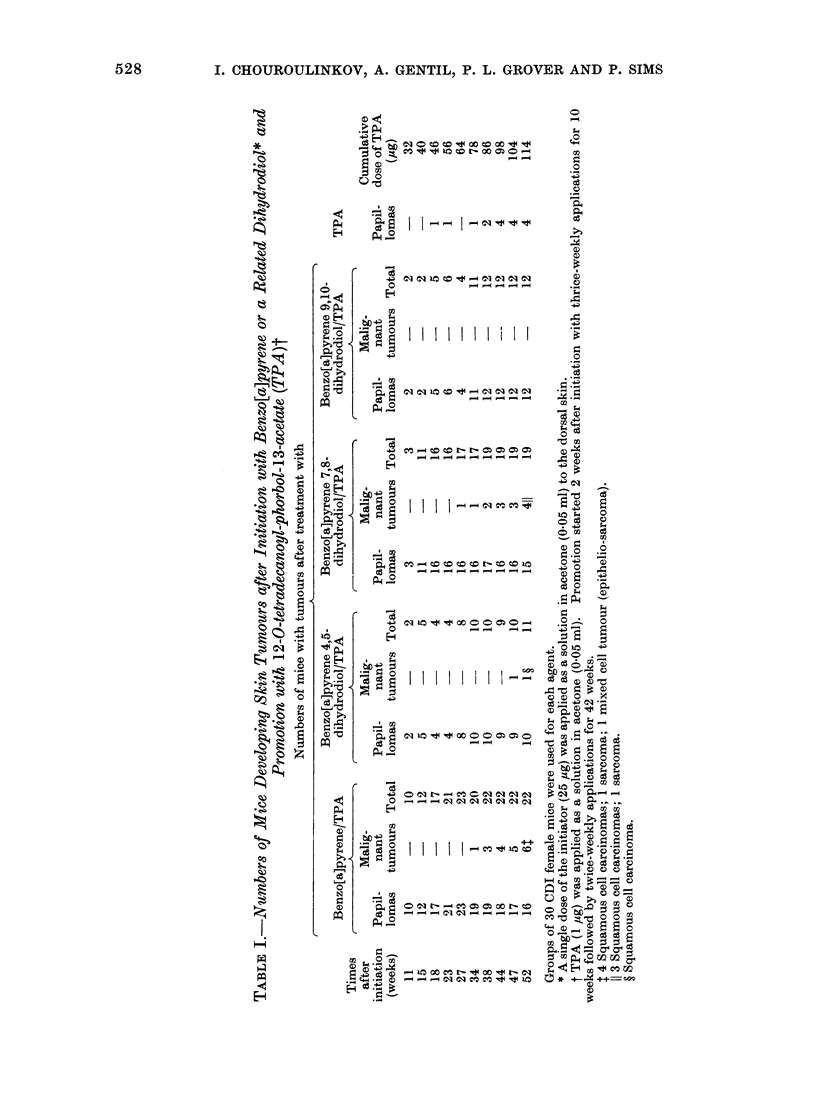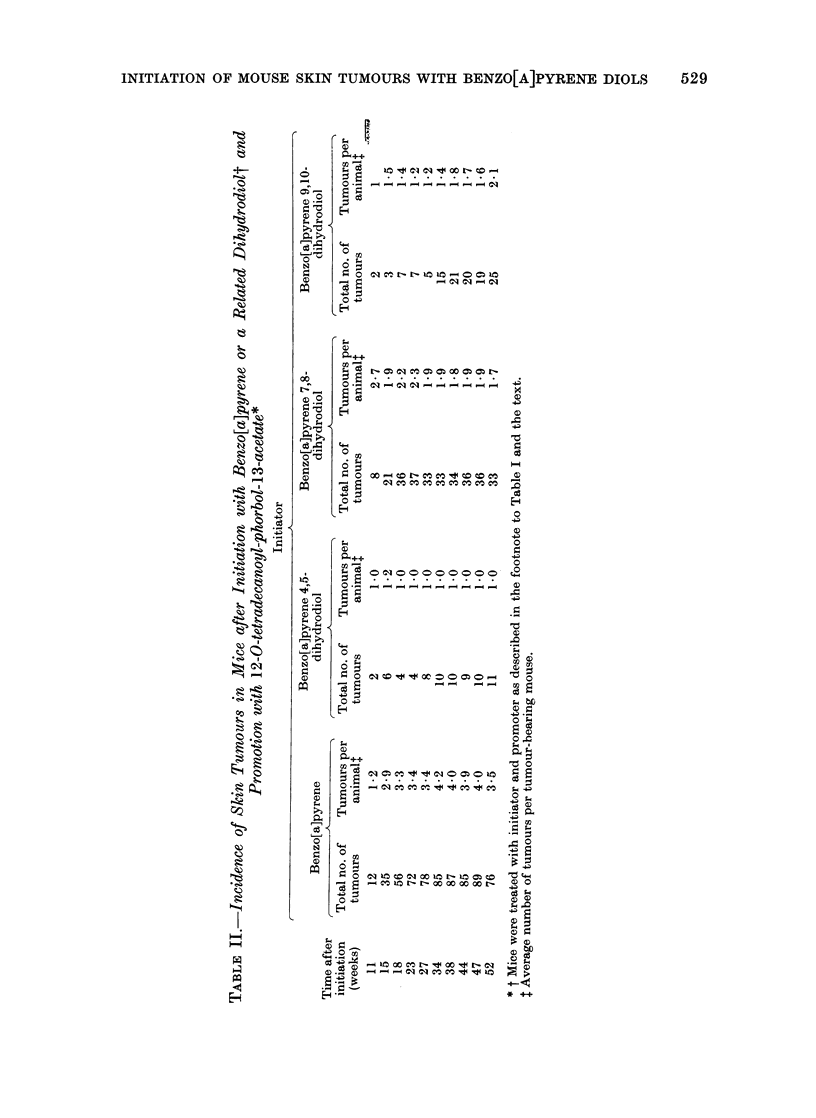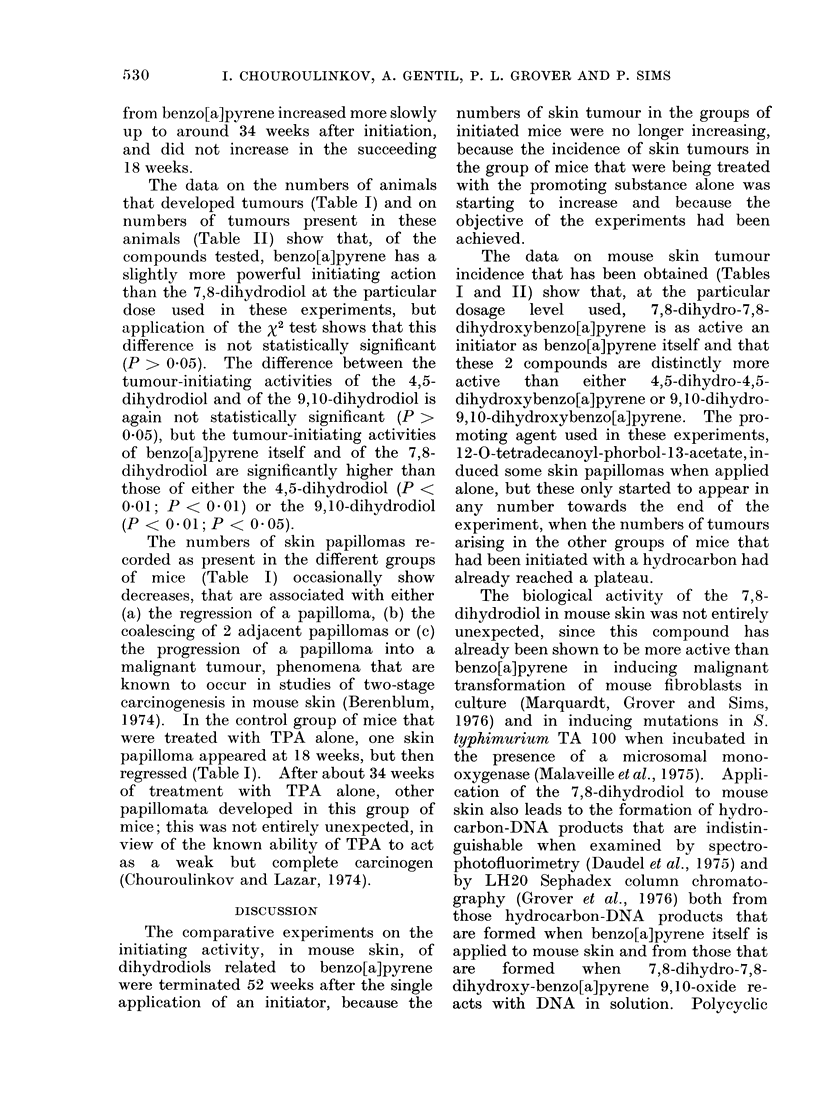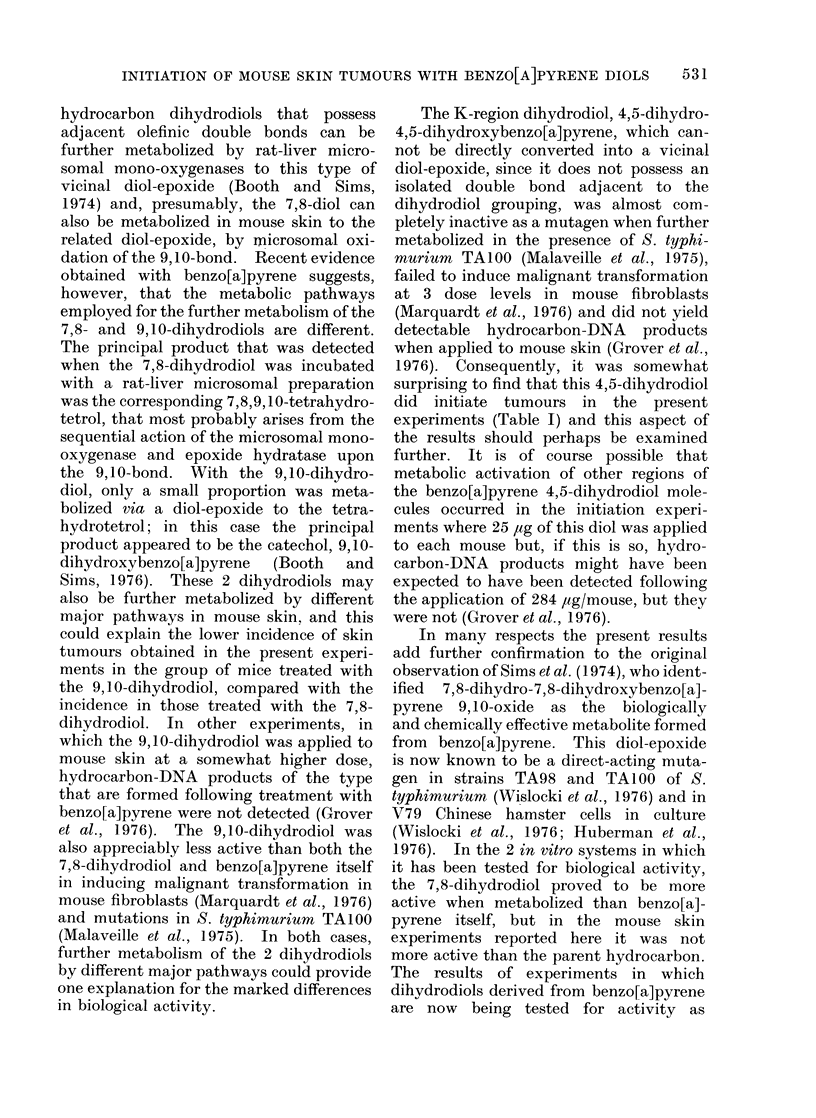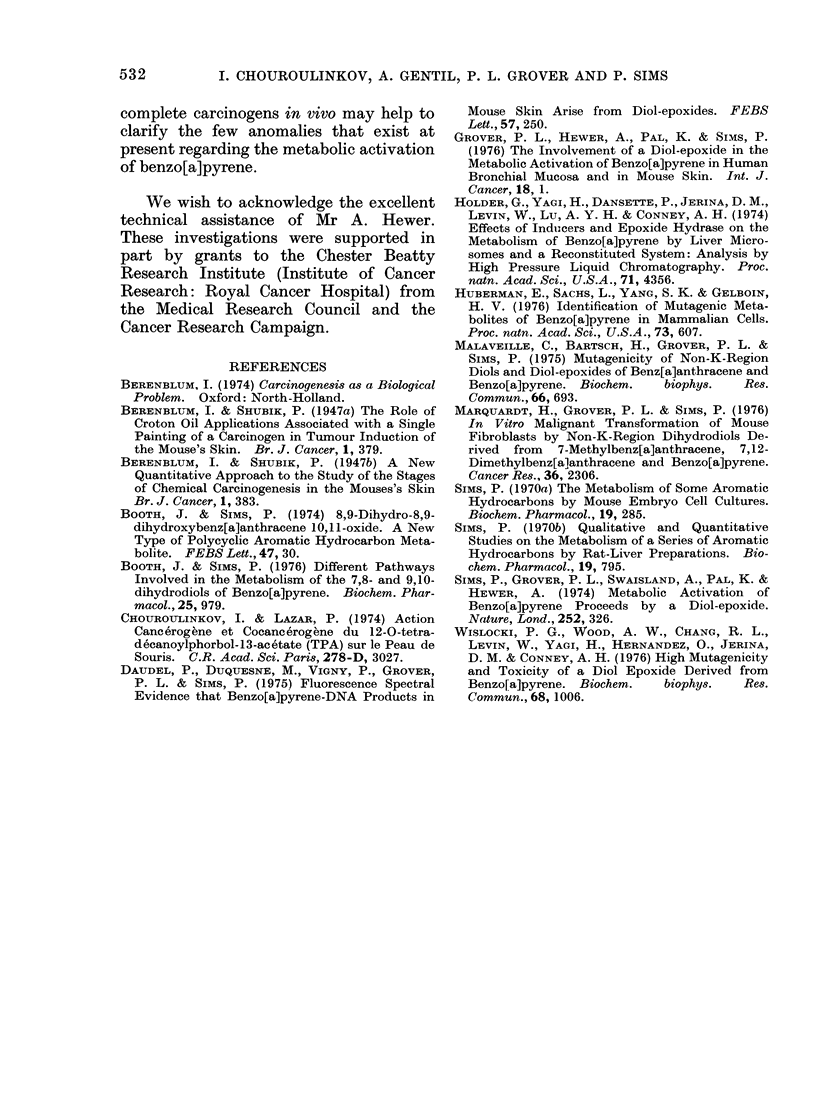# Tumour-initiating activities on mouse skin of dihydrodiols derived from benzo[a]pyrene.

**DOI:** 10.1038/bjc.1976.207

**Published:** 1976-11

**Authors:** I. Chouroulinkov, A. Gentil, P. L. Grover, P. Sims

## Abstract

**Images:**


*Br. J. Cancer* (1976) **34**, 523

### TUMOUR-INITIATING ACTIVITIES ON MOUSE SKIN OF DIHYDRODIOLS DERIVED FROM BENZO[a]PYRENE

I. CHOUROULINKOV,* A. GENTIL,* P. L. GROVER† AND P. SIMS†

*From *L'Institut de Recherches Scientifiques sur le Cancer, Boite Postale No.* 8,94800 *Villejuif, France and †The Chester Beatty Research Institute, Institute of Cancer Research, Royal Cancer Hospital, Fulham Road, London SW*3 6*JB, England*

Received 4 June 1976 Accepted 1 July 1976

**Summary.—Three dihydrodiols that are metabolites of benzo[a]pyrene and benzo[a]-pyrene itself have been tested in a comparative experiment for their activities as initiators of tumours in mouse skin. A single application (25 μg) of 4,5-dihydro-4,5-dihydroxybenzo[a]pyrene, of 7,8-dihydro-7,8-dihydroxybenzo[a]pyrene, of 9,10-dihydro-9,10-dihydroxybenzo[a]pyrene, or of benzo[a]pyrene was made to the shaved dorsal skin of adult female CDI mice; this was followed 2 weeks later by multiple thrice- or twice-weekly applications (1 μg) of 12-O-tetradecanoyl-phorbol-13-acetate as promoting agent. A control group of 30 mice received the promoting agent alone. The experiments were terminated 52 weeks after initiation. At this stage, all the groups contained mice bearing skin papillomas, some of which had progressed to malignancy. Quantitatively the results show that the 7,8-dihydrodiol is almost as active an initiator of mouse skin tumours as benzo[a]pyrene itself; the 4,5- and 9,10-dihydrodiols were significantly less active. The significance of these results is discussed in relation to the hypothesis that diol-epoxides are important in the metabolic activation of polycyclic hydrocarbons like benzo[a]pyrene.**

THE INITIAL EVIDENCE showing that the metabolic activation of benzo[a]pyrene (formula shown) involved the formation of 7,8-dihydro-7,8-dihydroxybenzo[a]pyrene 9,10-oxide was obtained with hamster embryo cells in culture (Sims *et al.*, 1974) and the mechanism appears to be similar in mouse skin. Thus, spectrophotofluorimetric studies indicated that metabolism of the 7,8,9,10-ring was involved in the activation of benzo[a]pyrene in mouse skin (Daudel *et al.*, 1975) and the chromatographic characteristics of the benzo[a]-pyrene-deoxyribonucleoside products that are formed in mouse skin treated with the hydrocarbon were recently found to be the same as those of the deoxyribonucleoside products that are formed when 7,8-dihydro-7,8-dihydroxybenzo[a]pyrene 9,10-oxide reacts with DNA in solution (Grover *et al.*, 1976). This is in agreement with the general idea that the further metabolism of the olefinic double bonds present in non-K-region dihydrodiols can yield diol-epoxides (Booth and Sims, 1974) that react with DNA *in vivo*. The biological activity of non-K-region dihydrodiols in situations where they can be further metabolized is, therefore, of interest. Some non-K-region dihydrodiols are more active in the induction of malignant transformation in mouse fibroblasts than the parent hydrocarbons (Marquardt, Grover and Sims, 1976) and they can also induce more mutations, when metabolized by microsomal preparations in the presence of *S. typhimurium* TA100 (Malaveille *et al.*, 1975).

One method of examining the carcinogenic activity of metabolites that are only available in small amounts is to test them for initiating activity on mouse skin that is then treated with a promoting agent (Berenblum and Shubik, 1947*a*, *b*; Berenblum, 1974). This paper presents the results that were obtained when benzo[a]pyrene and the related 4,5-, 7,8- and 9,10-dihydrodiols were tested for initiating activity by applying them, as a single dose, to the dorsal skin of adult, female, CDI mice; the appearance of tumours was promoted by subsequent multiple applications of 12-O-tetradecanoyl-phorbol-13-acetate.

### MATERIALS AND METHODS

Female 43-day-old CDI mice (Charles River, France) that had been vaccinated against ectromelia 14 days earlier, were randomized into groups of 30. The animals were then housed in individual cages for the duration of the experiments in order to prevent inter-mouse licking.

Benzo[a]pyrene (Sigma Chemical Co, St Louis, Mo, U.S.A.) was purified by column chromatography on alumina and recrystallization. The K-region dihydrodiol, *trans*-4,5-dihydroxybenzo[a]pyrene was prepared from the corresponding *cis*-isomer (Sims, 1970*a*). The non-K-region dihydrodiols, 7,8-dihydro-7,8-dihydroxybenzo[a]pyrene and 9,10-dihydro-9,10-dihydroxybenzo[a]pyrene, which were obtained, presumably as the *trans*-isomers, from large scale incubations of benzo[a]pyrene with rat-liver homogenates (Sims, 1970*b*), were purified by thin-layer chromatography on silica gel (Sims, 1970*b*) and by high-pressure liquid chromatography (Holder *et al.*, 1974) and were characterized by their chromatographic and u.v. spectral properties. 12-O-tetradecanoyl-phorbol-13-acetate (TPA) was very kindly donated by Professor E. Hecker, Heidelburg, Germany.

### *Treatment*

*Initiation.*—A single dose (25 $\mu$g) of benzo[a]pyrene, or of one of the three benzo[a]pyrene dihydrodiols, was applied as a solution in acetone (0·05 ml) to the dorsal skin of mice that had been closely clipped 48 h earlier.

*Promotion.*—Treatment with phorbol ester (TPA) was started 2 weeks after the application of the initiator. For the first 10 weeks of promotion, TPA (1 $\mu$g) was applied thrice weekly as a solution in acetone (0·05 ml) and, for the following 42 weeks, was applied twice weekly. The total dose of TPA applied to the dorsal skin of each mouse was 114 $\mu$g.

All the treatments with initiators and promoters were made with the aid of an accurate automatic microvolumetric dispenser. The animals were examined regularly, and the times of appearance of cutaneous tumours, both papillomas and malignant neoplasms, were recorded. Systematic postmortem and histological examinations were performed on all animals.

### RESULTS

### *Skin tumour morphology*

The tumours seen in the areas of treated skin were papillomas and malignant neoplasms (Fig. 1*a*). The papillomas were included in the results as soon as they were palpable and clearly visible. At this stage they were around 2–3 mm in diameter but they commonly reached 1 cm in diameter. In most cases, malignant transformation of papillomas was accompanied by ulceration (Fig. 1*a*).

Histologically, the papillomas showed an epidermal proliferation that was accompanied by hyperkeratinization (Fig. 1*b*). The malignant neoplasms were squamous cell carcinomas (Fig. 2 and 3*a*), which showed invasion of the muscle layer of the skin, and fibrosarcomas (Fig. 3*b*). One mixed-cell tumour, an epithelio-sarcoma, was also seen.

### *Skin tumour incidence*

Skin tumours started to appear about 10 weeks after initiation of the mice; detailed data on the time of tumour appearance and on the numbers of mice with tumours in each group are shown in Table I. The first papillomas appeared at about the same time in the 3 groups initiated with the different benzo[a]pyrene

FIG. 1 (*a*).—Macroscopic aspects of skin tumours induced after initiation with 7,8-dihydro-7,8-dihydroxybenzo[a]pyrene (Mice Nos. 10 and 12) and 9,10-dihydro-9,10-dihydroxybenzo[a]pyrene (Mouse No. 34) Papillomas (1), a squamous cell carcinoma (2) and a sarcoma (3) are present. (*b*) Double papilloma present on mouse skin (Mouse No. 12). There is no invasion of the muscle layer (stratum carnosum) (↑). × 25.

dihydrodiols but, with benzo[a]pyrene itself, they appeared somewhat earlier (Table I). In the groups of mice initiated with benzo[a]pyrene or with the 7,8-dihydrodiol, the tumour incidence subsequently increased sharply up to about 27 weeks after initiation, but did not increase thereafter (Table I). Skin tumour incidence in the mice initiated with the 4,5 and 9,10-dihydrodiols derived

FIG. 2.—Histology of a squamous cell carcinoma induced by 7,8-dihydro-7,8-dihydroxybenzo[a]-pyrene on mouse skin. (*a*) General appearance (× 40). (*b*) Higher magnification (× 100) of a portion of *a*, showing involvement of the muscle layer (↑). (The tumour is that shown on Mouse No. 10 in Fig. 1*a*).

FIG. 3 (*a*).—Dedifferentiated squamous cell carcinoma showing invasion of the stratum carnosum (× 100). Initiator: benzo[a]pyrene. (*b*). Subcutaneous fibroscarcoma (× 100). The tumour was initiated with 7,8-dihydro-7,8-dihydroxybenzo[a]pyrene and is that shown macroscopically on Mouse No. 12 in Fig. 1*a*.

§ Squamous cell carcinoma.

from benzo[a]pyrene increased more slowly up to around 34 weeks after initiation, and did not increase in the succeeding 18 weeks.

The data on the numbers of animals that developed tumours (Table I) and on numbers of tumours present in these animals (Table II) show that, of the compounds tested, benzo[a]pyrene has a slightly more powerful initiating action than the 7,8-dihydrodiol at the particular dose used in these experiments, but application of the $\chi^2$ test shows that this difference is not statistically significant ($P > 0{\cdot}05$). The difference between the tumour-initiating activities of the 4,5-dihydrodiol and of the 9,10-dihydrodiol is again not statistically significant ($P > 0{\cdot}05$), but the tumour-initiating activities of benzo[a]pyrene itself and of the 7,8-dihydrodiol are significantly higher than those of either the 4,5-dihydrodiol ($P < 0{\cdot}01$; $P < 0{\cdot}01$) or the 9,10-dihydrodiol ($P < 0{\cdot}01$; $P < 0{\cdot}05$).

The numbers of skin papillomas recorded as present in the different groups of mice (Table I) occasionally show decreases, that are associated with either (a) the regression of a papilloma, (b) the coalescing of 2 adjacent papillomas or (c) the progression of a papilloma into a malignant tumour, phenomena that are known to occur in studies of two-stage carcinogenesis in mouse skin (Berenblum, 1974). In the control group of mice that were treated with TPA alone, one skin papilloma appeared at 18 weeks, but then regressed (Table I). After about 34 weeks of treatment with TPA alone, other papillomata developed in this group of mice; this was not entirely unexpected, in view of the known ability of TPA to act as a weak but complete carcinogen (Chouroulinkov and Lazar, 1974).

### DISCUSSION

The comparative experiments on the initiating activity, in mouse skin, of dihydrodiols related to benzo[a]pyrene were terminated 52 weeks after the single application of an initiator, because the numbers of skin tumour in the groups of initiated mice were no longer increasing, because the incidence of skin tumours in the group of mice that were being treated with the promoting substance alone was starting to increase and because the objective of the experiments had been achieved.

The data on mouse skin tumour incidence that has been obtained (Tables I and II) show that, at the particular dosage level used, 7,8-dihydro-7,8-dihydroxybenzo[a]pyrene is as active an initiator as benzo[a]pyrene itself and that these 2 compounds are distinctly more active than either 4,5-dihydro-4,5-dihydroxybenzo[a]pyrene or 9,10-dihydro-9,10-dihydroxybenzo[a]pyrene. The promoting agent used in these experiments, 12-O-tetradecanoyl-phorbol-13-acetate, induced some skin papillomas when applied alone, but these only started to appear in any number towards the end of the experiment, when the numbers of tumours arising in the other groups of mice that had been initiated with a hydrocarbon had already reached a plateau.

The biological activity of the 7,8-dihydrodiol in mouse skin was not entirely unexpected, since this compound has already been shown to be more active than benzo[a]pyrene in inducing malignant transformation of mouse fibroblasts in culture (Marquardt, Grover and Sims, 1976) and in inducing mutations in *S. typhimurium* TA 100 when incubated in the presence of a microsomal monooxygenase (Malaveille *et al.*, 1975). Application of the 7,8-dihydrodiol to mouse skin also leads to the formation of hydrocarbon-DNA products that are indistinguishable when examined by spectrophotofluorimetry (Daudel *et al.*, 1975) and by LH20 Sephadex column chromatography (Grover *et al.*, 1976) both from those hydrocarbon-DNA products that are formed when benzo[a]pyrene itself is applied to mouse skin and from those that are formed when 7,8-dihydro-7,8-dihydroxy-benzo[a]pyrene 9,10-oxide reacts with DNA in solution. Polycyclic hydrocarbon dihydrodiols that possess adjacent olefinic double bonds can be further metabolized by rat-liver microsomal mono-oxygenases to this type of vicinal diol-epoxide (Booth and Sims, 1974) and, presumably, the 7,8-diol can also be metabolized in mouse skin to the related diol-epoxide, by microsomal oxidation of the 9,10-bond. Recent evidence obtained with benzo[a]pyrene suggests, however, that the metabolic pathways employed for the further metabolism of the 7,8- and 9,10-dihydrodiols are different. The principal product that was detected when the 7,8-dihydrodiol was incubated with a rat-liver microsomal preparation was the corresponding 7,8,9,10-tetrahydrotetrol, that most probably arises from the sequential action of the microsomal mono-oxygenase and epoxide hydratase upon the 9,10-bond. With the 9,10-dihydrodiol, only a small proportion was metabolized *via* a diol-epoxide to the tetrahydrotetrol; in this case the principal product appeared to be the catechol, 9,10-dihydroxybenzo[a]pyrene (Booth and Sims, 1976). These 2 dihydrodiols may also be further metabolized by different major pathways in mouse skin, and this could explain the lower incidence of skin tumours obtained in the present experiments in the group of mice treated with the 9,10-dihydrodiol, compared with the incidence in those treated with the 7,8-dihydrodiol. In other experiments, in which the 9,10-dihydrodiol was applied to mouse skin at a somewhat higher dose, hydrocarbon-DNA products of the type that are formed following treatment with benzo[a]pyrene were not detected (Grover *et al.*, 1976). The 9,10-dihydrodiol was also appreciably less active than both the 7,8-dihydrodiol and benzo[a]pyrene itself in inducing malignant transformation in mouse fibroblasts (Marquardt *et al.*, 1976) and mutations in *S. typhimurium* TA100 (Malaveille *et al.*, 1975). In both cases, further metabolism of the 2 dihydrodiols by different major pathways could provide one explanation for the marked differences in biological activity.

The K-region dihydrodiol, 4,5-dihydro-4,5-dihydroxybenzo[a]pyrene, which cannot be directly converted into a vicinal diol-epoxide, since it does not possess an isolated double bond adjacent to the dihydrodiol grouping, was almost completely inactive as a mutagen when further metabolized in the presence of *S. typhimurium* TA100 (Malaveille *et al.*, 1975), failed to induce malignant transformation at 3 dose levels in mouse fibroblasts (Marquardt *et al.*, 1976) and did not yield detectable hydrocarbon-DNA products when applied to mouse skin (Grover *et al.*, 1976). Consequently, it was somewhat surprising to find that this 4,5-dihydrodiol did initiate tumours in the present experiments (Table I) and this aspect of the results should perhaps be examined further. It is of course possible that metabolic activation of other regions of the benzo[a]pyrene 4,5-dihydrodiol molecules occurred in the initiation experiments where 25 $\mu$g of this diol was applied to each mouse but, if this is so, hydrocarbon-DNA products might have been expected to have been detected following the application of 284 $\mu$g/mouse, but they were not (Grover *et al.*, 1976).

In many respects the present results add further confirmation to the original observation of Sims *et al.* (1974), who identified 7,8-dihydro-7,8-dihydroxybenzo[a]-pyrene 9,10-oxide as the biologically and chemically effective metabolite formed from benzo[a]pyrene. This diol-epoxide is now known to be a direct-acting mutagen in strains TA98 and TA100 of *S. typhimurium* (Wislocki *et al.*, 1976) and in V79 Chinese hamster cells in culture (Wislocki *et al.*, 1976; Huberman *et al.*, 1976). In the 2 *in vitro* systems in which it has been tested for biological activity, the 7,8-dihydrodiol proved to be more active when metabolized than benzo[a]-pyrene itself, but in the mouse skin experiments reported here it was not more active than the parent hydrocarbon. The results of experiments in which dihydrodiols derived from benzo[a]pyrene are now being tested for activity as complete carcinogens *in vivo* may help to clarify the few anomalies that exist at present regarding the metabolic activation of benzo[a]pyrene.

We wish to acknowledge the excellent technical assistance of Mr A. Hewer. These investigations were supported in part by grants to the Chester Beatty Research Institute (Institute of Cancer Research: Royal Cancer Hospital) from the Medical Research Council and the Cancer Research Campaign.

### REFERENCES

BERENBLUM, I. (1974) *Carcinogenesis as a Biological Problem.* Oxford: North-Holland.

BERENBLUM, I. & SHUBIK, P. (1947*a*) The Role of Croton Oil Applications Associated with a Single Painting of a Carcinogen in Tumour Induction of the Mouse's Skin. *Br. J. Cancer*, **1**, 379.

BERENBLUM, I. & SHUBIK, P. (1947*b*) A New Quantitative Approach to the Study of the Stages of Chemical Carcinogenesis in the Mouses's Skin *Br. J. Cancer*, **1**, 383.

BOOTH, J. & SIMS, P. (1974) 8,9-Dihydro-8,9-dihydroxybenz[a]anthracene 10,11-oxide. A New Type of Polycyclic Aromatic Hydrocarbon Metabolite. *FEBS Lett.*, **47**, 30.

BOOTH, J. & SIMS, P. (1976) Different Pathways Involved in the Metabolism of the 7,8- and 9,10-dihydrodiols of Benzo[a]pyrene. *Biochem. Pharmacol.*, **25**, 979.

CHOUROULINKOV, I. & LAZAR, P. (1974) Action Cancérogène et Cocancérogène du 12-O-tetradécanoylphorbol-13-acétate (TPA) sur le Peau de Souris. *C.R. Acad. Sci. Paris*, **278-D**, 3027.

DAUDEL, P., DUQUESNE, M., VIGNY, P., GROVER, P. L. & SIMS, P. (1975) Fluorescence Spectral Evidence that Benzo[a]pyrene-DNA Products in Mouse Skin Arise from Diol-epoxides. *FEBS Lett.*, **57**, 250.

GROVER, P. L., HEWER, A., PAL, K. & SIMS, P. (1976) The Involvement of a Diol-epoxide in the Metabolic Activation of Benzo[a]pyrene in Human Bronchial Mucosa and in Mouse Skin. *Int. J. Cancer*, **18**, 1.

HOLDER, G., YAGI, H., DANSETTE, P., JERINA, D. M., LEVIN, W., LU, A. Y. H. & CONNEY, A. H. (1974) Effects of Inducers and Epoxide Hydrase on the Metabolism of Benzo[a]pyrene by Liver Microsomes and a Reconstituted System: Analysis by High Pressure Liquid Chromatography. *Proc. natn. Acad. Sci., U.S.A.*, **71**, 4356.

HUBERMAN, E., SACHS, L., YANG, S. K. & GELBOIN, H. V. (1976) Identification of Mutagenic Metabolites of Benzo[a]pyrene in Mammalian Cells. *Proc. natn. Acad. Sci., U.S.A.*, **73**, 607.

MALAVEILLE, C., BARTSCH, H., GROVER, P. L. & SIMS, P. (1975) Mutagenicity of Non-K-Region Diols and Diol-epoxides of Benz[a]anthracene and Benzo[a]pyrene. *Biochem. biophys. Res. Commun.*, **66**, 693.

MARQUARDT, H., GROVER, P. L. & SIMS, P. (1976) *In Vitro* Malignant Transformation of Mouse Fibroblasts by Non-K-Region Dihydrodiols Derived from 7-Methylbenz[a]anthracene, 7,12-Dimethylbenz[a]anthracene and Benzo[a]pyrene. *Cancer Res.*, **36**, 2306.

SIMS, P. (1970*a*) The Metabolism of Some Aromatic Hydrocarbons by Mouse Embryo Cell Cultures. *Biochem. Pharmacol.*, **19**, 285.

SIMS, P. (1970*b*) Qualitative and Quantitative Studies on the Metabolism of a Series of Aromatic Hydrocarbons by Rat-Liver Preparations. *Biochem. Pharmacol.*, **19**, 795.

SIMS, P., GROVER, P. L., SWAISLAND, A., PAL, K. & HEWER, A. (1974) Metabolic Activation of Benzo[a]pyrene Proceeds by a Diol-epoxide. *Nature, Lond.*, **252**, 326.

WISLOCKI, P. G., WOOD, A. W., CHANG, R. L., LEVIN, W., YAGI, H., HERNANDEZ, O., JERINA, D. M. & CONNEY, A. H. (1976) High Mutagenicity and Toxicity of a Diol Epoxide Derived from Benzo[a]pyrene. *Biochem. biophys. Res. Commun.*, **68**, 1006.